# Synchronous Sampling-Based Direct Current Estimation Method for Self-Sensing Active Magnetic Bearings

**DOI:** 10.3390/s20123497

**Published:** 2020-06-20

**Authors:** Xiongxin Hu, Fang Xu, Ronghui Wang, Dapeng Tan

**Affiliations:** 1 College of Mechanical Engineering, Zhejiang University of Technology, Hangzhou 310014, China; huxx007@zjut.edu.cn (X.H.); fangx@zjut.edu.cn (F.X.); 2111802073@zjut.edu.cn (R.W.); 2 Key Laboratory of Special Purpose Equipment and Advanced Processing Technology, Ministry of Education and Zhejiang Province, Zhejiang University of Technology, Hangzhou 310014, China; 3 Collaborative Innovation Center of High-end Laser Manufacturing Equipment (National “2011 Plan”), Zhejiang University of Technology, Hangzhou 310014, China

**Keywords:** self-sensing, active magnetic bearings, least square method, direct current estimator, synchronous sampling

## Abstract

Active magnetic bearings (AMBs) commonly use pulse-width modulation to reduce analogous hardware and manufacturing costs, but they experience sensing process, sensing accuracy and stability problems. To address these issues, a synchronous sampling-based direct current estimation (SS-DCE) method is proposed herein with a bistate switching power amplifier. First—considering the reluctance evolution mechanism of AMBs—a coupling relation mathematical model between rotor displacement and voltage/current is presented to acquire the rotor position from the working coil current alone. Then—assuming that the switching current was an approximately triangular signal—a DCE for the rotor position was established based on the estimation inductance of the charging/discharging phase. Finally—to decrease the phase shift caused by the self-sensing filters and position estimation algorithms—the SS-DCE method was introduced to conduct precise position detection for rotors with high velocities. The simulation and experimental results indicated that the proposed method could improve the sensing accuracy and stability. Compared to other AMB position estimation methods, the simple linearity of the SS-DCE method was greatly improved and could be controlled below 4%. Evaluation using frequency response analysis showed that the SS-DCE method had excellent dynamic accuracy and could perform at a higher phase margin, especially for the uprising/landing transient state. Moreover, there was a phase margin of 158° at the natural frequency of 19.26 HZ, and the peak sensitivity in the 50–250 μm range reached 10.7 dB.

## 1. Introduction

**Figure 1:**
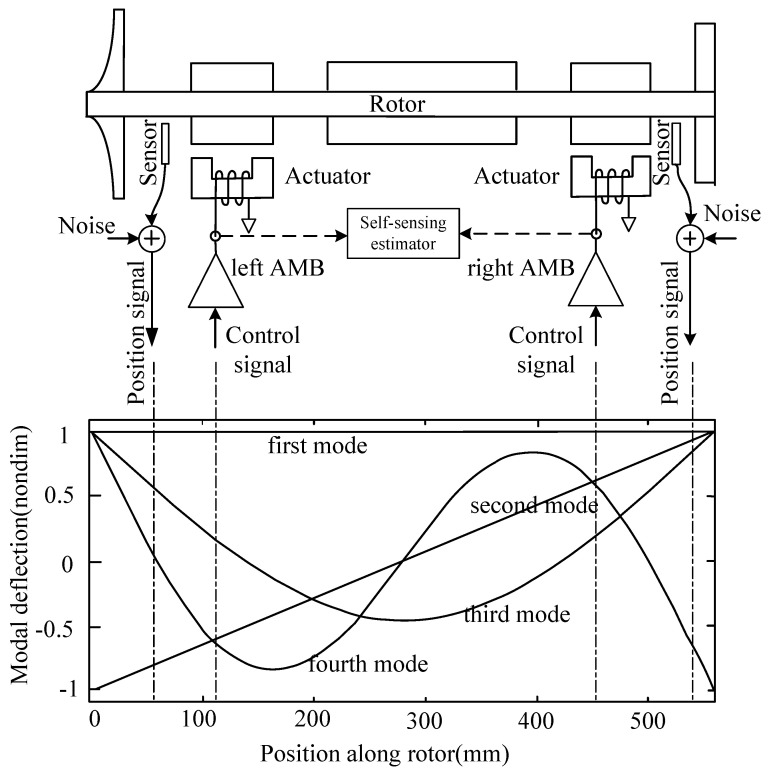
Example of a generic active magnetic bearing (AMB) system and free–free rotor mode.

For the rotating machinery of pumps, generators, turbomachines or machine tools, the rotors are generally supported by hydraulic bearings, composite ceramic bearings or magnetic bearings. Due to their high-speed rotation, high efficiency and low maintenance demand, active magnetic bearings (AMBs) have been applied in a series of modern industrial high-speed motor systems to replace conventional bearings. However, their geometric size, manufacturing cost and work reliability are disadvantageous as they comprise numerous components, such as wires, electromagnetic actuators, sensors and controllers. If some of these components are eliminated or integrated, the cost and size can be reduced, and the system reliability can be improved. The first self-sensing method proposed by Vischer [1] is based on the Luenberger model, but its estimation results are sensitive to systematic parameter changes. However, Cannon et al. [2] and Karkoub et al. [3] found that stable control is easy to achieve in flexible structures with co-located sensors and actuators. For example, a generic AMB-supported rotor, peripheral hardware and free–free rotor mode [4] are shown in Figure 1. This AMB structure is non-collocated because the position sensor is set beside the actuator (electromagnet); the phase difference between the position sensor and the actuator can be potentially dangerous if the rotor is running in a high-order mode. Then, the co-located solution, called self-sensing method, is developed, and the phase difference will be eliminated because the position sensor and the actuator will combine.

Self-sensing methods can be divided into two main categories: state observer-based and parameter estimator–based methods. Vischer [5] and Mizuno [6] presented the rotor position as a system state, but the system robustness based on the observer was lacking in comparison to that based on the sensor. In 1998, Morse [7] first proposed that the design scheme of self-sensing bearings lacks robustness to the bearing model’s uncertainty; hence, several parameter estimators were developed. A high-frequency small dither signal is injected into the coil of AMBs because Maslen [8] showed that the linear period signal can increase robustness. The inductance change was detected and the rotor position was estimated by Park et al. [9], Ghule et al. [10], Bugsch et al. [11] and Tan et al. [12]. However, this approach is limited by the bandwidth of the power amplifier (PA) and the signal-to-noise ratio. Another solution based on the ripples of a pulse–width modulation (PWM) PA introduced by Okada [13] and Noh [14] showed that the PWM ripple components are demodulated to estimate the rotor position, because the working current and voltage of AMBs are related to the inductance of the bearing coil. To overcome the disadvantage of the duty cycle of the PWM PA current not being fixed and affecting the detection accuracy, Mizuno [15] used a hysteresis amplifier to drive the magnetic bearing and converted the rotor displacement to the switching frequency of the hysteresis amplifier. Furthermore, Schammass [16] suggested that digital filtering amplitude demodulation (DFAD) is better than Noh’s analog approach.

**Figure 2:**
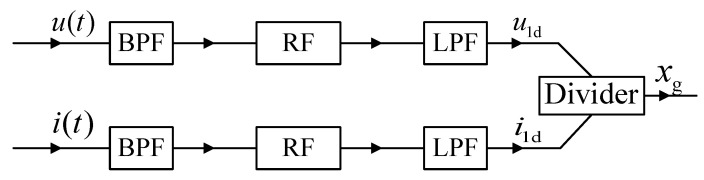
Diagram of the AFAD approach.

**Figure 3:**
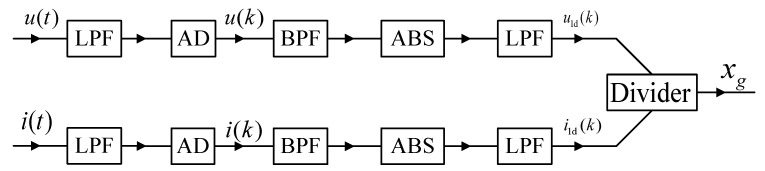
Diagram of the digital filtering amplitude demodulation (DFAD) approach.

The analog solution of Schammass’s approach is shown in Figure 2, while its digital solution is shown in Figure 3. Both solutions inherently involve the band-pass filter (BPF), rectifier or absolution algorithm and low-pass filter (LPF) to isolate the fundamental ripples of the coil current and voltage. Lu et al. [17], Zhang et al. [18,19], Pan et al. [20] and Tan et al. [21,22] investigated the digital model of amplitude demodulation. Similarly, Yu et al. [23] and Li et al [24,25] improved the estimation accuracy using an accurate analytical model in the frequency domain. Moreover, the displacement estimation module of the amplitude demodulation method has been constructed, including BPF, LPF and envelope detection module. Compared to the analog filtering amplitude demodulation (AFAD), DFAD increases the ADC (analog-to-digital converter) and replaces the analog filters with digital filters. Therefore, the outputs of the two estimators roughly have the same gain and phase response. The advantage of the amplitude demodulation estimation of both approaches is that it can conduct relatively mature electronic technology and involve communication demodulation method.

In general, the extra phase shift is inevitably introduced in the demodulator self-sensing process, comprising the signal conditioning the electronic circuits, the filters extracting the fundamental ripple and the position estimation algorithm operation. Another issue is the nonlinear effect of the change of the duty cycle, by which the estimation accuracy, stability and robustness will inevitably decrease.

**Figure 4:**
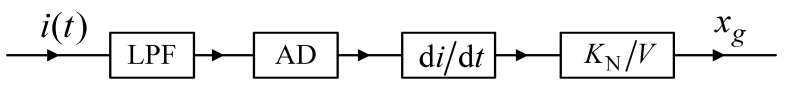
Diagram of the current gradient demodulation approach.

Another position estimation approach is gradient demodulation, which uses the coil current gradient signal. Li et al. [26] presented a self-sensing model based on the current change rate and Haarnoja et al. [27], Tian et al. [28] and Zhang et al. [29,30] reported that the rotor position can be estimated by the current slope due to the switching amplifier. This approach is obviously advantageous as no filters are employed in the self-sensing process and the nonlinear compensation of the duty cycle change effect is eliminated. Figure 4 shows the diagram of the gradient demodulation estimator. After the signal passes the anti-alias LPF, the demodulation method extracts the position information from the current’s rate of change (di/dt) or the gradient. In the figure, *K*_N_ is the product of the coil length and the self-inductance, *V* is the voltage, *i* is the current and t is the time.

Since the current sampling method and technique are limited by the nonlinear magnetic effect and the eddy current in the PA transients, several challenges exist in the precise measurement of the current slope. Hence, Glück et al. [31] and Rizzello et al. [32,33] proposed a rotor position calculated by the estimation inductance based on the least squares identification model with the coil current. Evidently, a large phase shift is introduced by a more complex algorithm of position estimation and the ADC with higher speed and greater precision is adopted in the self-sensing process [34]. The extra phase shift introduced by the complex estimation algorithm reported by Nevaranta et al. [35], Sun et al. [36], Yang et al. [37] and Ge et al. [38] or the advanced filters proposed by Sun et al. [39] and Matsuda et al. [40] will affect the stability and robustness of AMBs. Consequently, developing a technical solution scheme with an adequate phase margin for the long-term operation of self-sensing AMBs is necessary.

As the switching frequency is generally high, updating the rotor position at each switching cycle is difficult. Therefore, Ranft et al. [41], Van Schoor et al. [42] and Yu et al. [43] and Li et al. [44] suggested the reduction of the switching frequency or updating of the position every several cycles. Apparently, for self-sensing AMBs to adapt to high-speed rotary machines is challenging.

**Figure 5:**
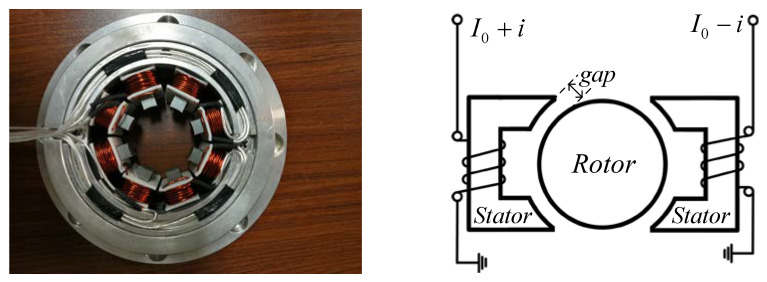
Eight-pole heteropolar radial stator (**left**) and single-degree-of-freedom (DOF) model of AMB (**right**).

If no flux cross-coupling is present between adjacent poles of the eight-pole heteropolar radial AMB, we can set up a principle model of a single-degree-of-freedom (DOF) AMB, as shown in Figure 5. Based on the model, the self-sensing solution employs an estimator that uses the coil current and voltage to calculate the gap between the rotor and stator [45]. The self-sensing estimator tends to utilize filters to extract the target signal, and its digital implementation must use the AD convector. Finite impulse response (FIR) filters or electronic filters are adopted in the self-sensing process. Since the external phase shift is introduced by the filters and complex estimation algorithms, the system stability margin will be limited. The symmetrical design of the demodulation circuit is the key section that concurrently takes charge of the fundamental current and voltage extraction process. Consequently, PWM switch amplifiers based on self-sensing schemes experience sensing process, sensing accuracy and work stability problems.

To address this issue, Schammass [16] proposed a normal ADC applied with the analog solution because the synchronous demodulator’s output is the fundamental ripple of the voltage and current. It is an inevitable consequence of the large phase shift introduced by the BPF and LPF in Niemann’s work [46]. Therefore, the temperature sensitivity and phase shift can be improved in the digital solution; another advantage of this solution is that changing the mathematical algorithm is easy [47,48]. The stability margin of the system will be limited since an extra phase shift is introduced by the FIR filters and complex estimation algorithms [11]. In addition, the symmetrical design of the demodulation plays a key role in the position estimator [49].

To address the above problems, this study proposes a position estimation method that can not only extract the rotor position from the current gradient information, but also reduce the overhead time of the estimation algorithm’s operation. A direct current estimator based on the least squares method (DCE-LSM) is introduced, and since the overhead time is too large, a synchronous sampling-based direct current estimator (SS-DCE) method is subsequently presented. The most obvious feature of the SS-DCE is the current sampling instant that is taken at the synchronized triangle wave of PWM in the bistate switching PA.

The rest of this study is organized as follows: Section 2 presents the position estimation method for direct current data. Section 3 discusses the simulation/experimental results and checks the static/dynamic performances of the proposed self-sensing estimator. Finally, in Section 4, the conclusions are summarized, and a short future outlook is mentioned.

## 2. Methodology

### 2.1. Research Framework

**Figure 6:**
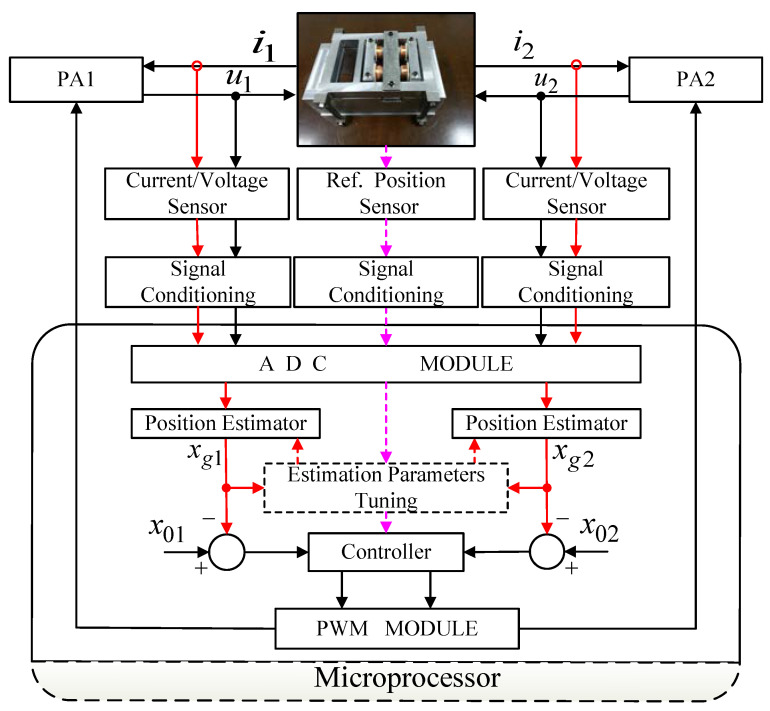
Research framework of the self-sensing AMBs.

To restrain the phase shift in the position estimator and improve the system performance and stability, a self-sensing research framework is proposed herein (Figure 6). In step 1, the closed-loop controller with a reference position sensor is set up, and the systematic parameters, which comprise the bias current, the current/position signal amplifier’s gain, the anti-alias filters, the PID controller parameters, etc., are tested and recorded. Then, the basic work framework of the AMBs is implemented. In step 2, the self-sensing framework is applied in the AMBs. Compared to the ideal sensor, the position estimators’ performances are measured in a simulation test. The position estimation algorithms include the SS-DCE, DCE-LSM, AFAD and direct current measurement (DCM) methods. In step 3, the experimental results are obtained on a single-DOF rig.

### 2.2. Amplitude Demodulation Algorithm

**Figure 7:**
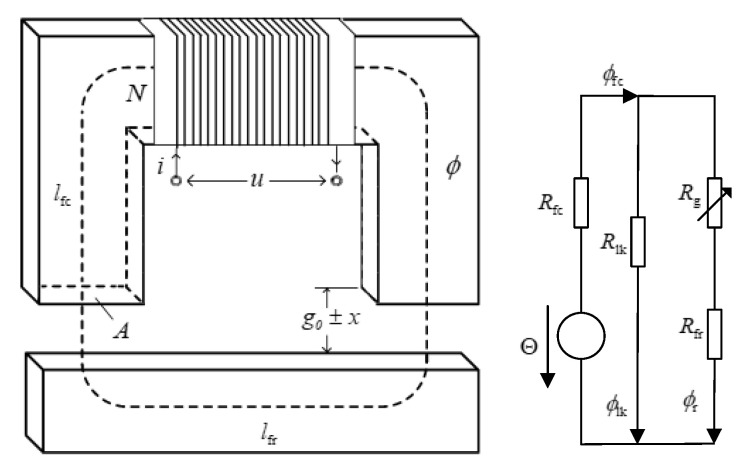
Reluctance model of single-DOF AMB.

In Figure 7, neglecting the nonlinear magnetic effects (i.e., hysteresis, saturation, eddy current and fringing) and flux leakage, the equivalent reluctance $R_{m}$ of the overall system is described as

(1) $$\begin{array}{c} R_{m}=R_{\mathrm{fc}}+R_{g}+R_{\mathrm{fr}}=\frac{1}{\mu_{0}A}\left[2(g_{0}\pm x)+\left(l_{\mathrm{fc}}+l_{\mathrm{fr}}\right)/\mu_{r}\right]\\ x_{g}=2(g_{0}\pm x)+\left(l_{\mathrm{fc}}+l_{\mathrm{fr}}\right)/\mu_{r}, \end{array}$$

where $R_{\mathrm{fc}}$ is the effective reluctance of the core, $R_{\mathrm{fr}}$ is the reluctance of the rotor, $R_{g}$ is the reluctance of the gap, $R_{\mathrm{lk}}$ is the reluctance of the leakage fluxes, *l*_fr_ and *l*_fc_ are the average length of the magnetic circuit of the iron rotor and core, respectively, *x* is the change in the air gap, *g*_0_ is the initial air gap length, *A* is the area of the magnetic pole, *l*_lk_ is the length of the magnetic flux leakage circuit, *μ*_0_ and *μ_r_* are the permeability of air and ferromagnetic materials, respectively and *x*_g_ is the estimation position amplitude.

According to the Faraday laws of inductance, ampere loop and flux conservation, we can obtain the model control equation as follows:

(2) $$u-iR=N^{2}\frac{d(i/R_{m})}{dt}=L(x_{g})\frac{di}{dt}+i\cdot \frac{\partial L(x_{g})}{\partial x_{g}}\frac{dx_{g}}{dt},$$

where $L=L(x_{g})=K_{N}/x_{g}$, $K_{N}=\mu_{0}N^{2}A$, u is the working voltage of the coil, i is the working current, *N* is the number of individual coil turns, and *R* is the electrical resistance. Then, the gradient of the current can be expressed as

(3) $$\frac{di}{dt}=\frac{1}{L(x_{g})}\left(u-iR-i\cdot \frac{\partial L(x_{g})}{\partial x_{g}}\frac{dx_{g}}{dt}\right).$$

Assuming that the rotor vibration is slow compared to the high-frequency coil current and that the coil resistance is far less than the coil inductive impedance, we can neglect the derivative term of the rotor position and coil resistance. The rotor position is described by

(4) $$x_{g}(t)=\frac{K_{N}}{2u}\frac{di}{dt}.$$

The integral of Equation (4) can be written as

(5) $$i=\frac{2x_{g}(t)}{K_{N}}\int udt.$$

If the switching fundamental ripple $i_{1}(t)=i_{1d}\cos(\omega_{s}t)$ and $u_{1}(t)=u_{1d}\sin(\omega_{s}t)$, the estimation position amplitude is expressed as

(6) $$x_{g}=\omega_{s}K_{N}\cdot \frac{i_{1d}}{u_{1d}}=K_{A}\cdot \frac{i_{1d}}{u_{1d}}.$$

Equation (6) is the conventional solution of the position estimation approach [16,50,51] as the amplitude demodulation approach, wherein $\omega_{s}$ is the angular frequency.

The DCM approach reported by Niemann [46] is a simplistic solution that measures the maximum amplitude of the current fundamental ripple isolated by BPF during a constant 50% duty cycle. The position estimation of DCM is depicted as

(7) $$x_{g}=\frac{-2}{K_{N}\omega_{s}}\left[\max(i_{1}(t)-\mathrm{avg}(i_{1}(t)))\right].$$

In the DCM estimation, the current ripple amplitude is measured with a constant 50% duty cycle each time; thus, voltage *u* in Equation (3) is constant. Since the switching time is now also fixed, the current gradient becomes proportional to the current amplitude during the measurement cycle.

### 2.3. DCE-LSM Algorithm

The switching current of PA is controlled by a duty cycle, and the flux of AMBs is always regulated by the switching current. Assuming that the rotor vibration position is represented by a sinusoid function $x_{m}\sin\omega_{c}t$, the duty cycle’s function can be described as $\alpha_{k}=\alpha_{0}\pm \alpha_{mk}\cos\omega_{c}t$ [14]. Meanwhile, the cosine function [51,52,53,54] of the bistate switching voltage and current of the electromagnets in the *k*th cycle can be expressed as follows:

(8) $$u_{k}(t)=V(2\alpha_{0}-1)+2V\alpha_{mk}\cos\left(\omega_{c}t\right)+\sum_{n=1}^{\infty }\frac{4V}{n\pi }\left|\sin(n\pi \alpha_{k})\right|\cos(n\omega_{s}t-n\pi \alpha_{k})$$

(9) $$i_{k}(t)=\frac{V(2\alpha_{0}-1)}{R}+\frac{2V\alpha_{mk}\cos\left(\omega_{c}t\right)}{\sqrt{R^{2}+{\left(\omega_{c}L\right)}^{2}}}+\sum_{n=1}^{\infty }\frac{4V\left|\sin(n\pi \alpha_{k})\right|}{n^{2}\pi \omega_{s}L}\cos(n\omega_{s}t-n\pi \alpha_{k})$$

where $\alpha_{0}$ is a constant for the duty cycle of the bias flux, $\alpha_{mk}$ is the quantity variation, *V* is the supply voltage of PA, $\omega_{c}$ is the control angular frequency, $\omega_{s}$ is the switching angular frequency and $\alpha_{k}\in (0,1)$.

**Figure 8:**
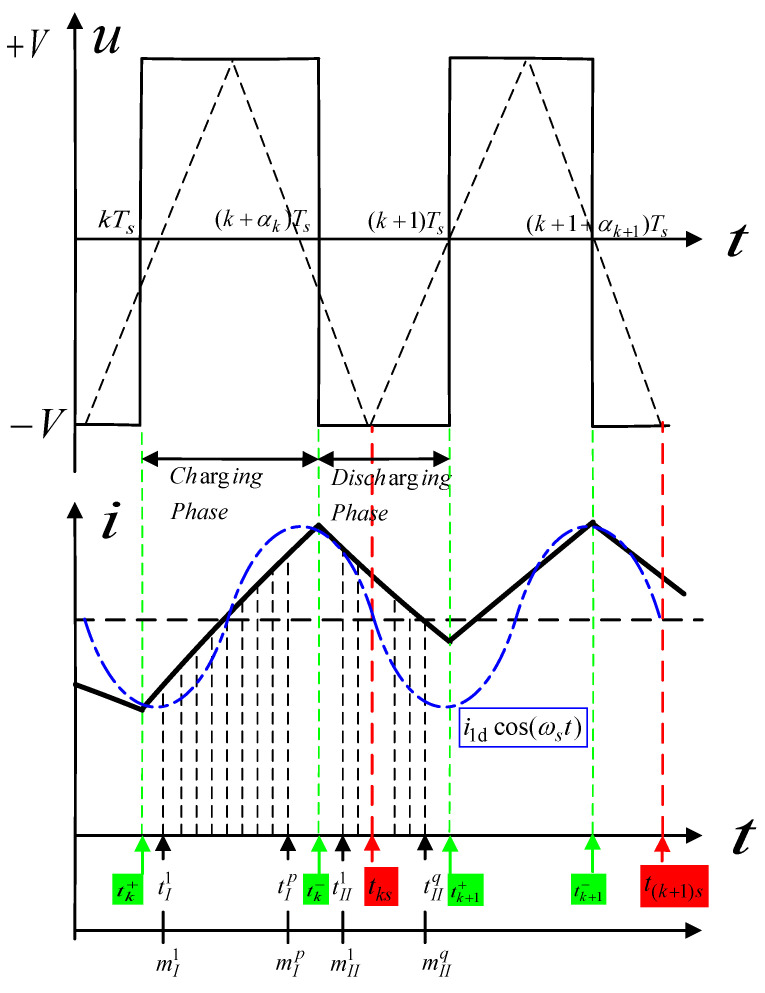
Switching voltage, current and sampling process.

In Figure 8, the switching voltage and current are shown, and the triangle carrier is drawn as a symmetrical wave that is compared with the reference signal controlling the PWM generator. Avoiding the influence of the switching glitches, the current measurement occurs in the time interval $\left[t_{I}^{1},t_{I}^{p}\right]$. The current and change of flux linkage in the charging phase can be described as

(10) $$i(k_{I})=i(t_{I}^{1})+\frac{1}{L_{k_{I}}}\int_{t_{I}^{1}}^{k_{I}}\left(u-Ri\right)dt$$

(11) $$\Delta \psi_{k_{I}}=\int_{t_{I}^{1}}^{k_{I}}\left(u-Ri\right)dt$$

where $\Delta \psi_{k_{I}}$ is $\Delta \psi (k_{I}T_{s})$ with $k_{I}=m_{I}^{1},\ldots ,m_{I}^{p}$ and sampling interval $T_{sp}$.

Then, the change of the discretized flux linkage is given by

(12) $$\begin{array}{l} \Delta \psi_{k_{I}}=T_{s}\sum_{j=m_{I}^{1}}^{k_{I}-1}\left(u_{j}-Ri_{j}\right)\\ \Delta \psi_{m_{s}^{I}}=0 \end{array}$$

where $k_{I}=m_{I}^{1}+1,\ldots ,m_{I}^{p}$. The current in Equation (10) reads as

(13) $$i_{k_{I}}=i_{m_{I}^{1}}+\frac{\Delta \psi_{k_{I}}}{L_{k_{I}}}.$$

Since the measurement noise will lead to very imprecise estimations, the resulting equations cannot be practically resolved. According to Equation (13), the inductance in the charging phase can be theoretically deduced in vector notation, wherein a quadratic measure is used by LSM.

(14) $$\underset{I_{mI}}{\underbrace{\left[\begin{array}{c} i_{m_{I}^{1}}\\ i_{m_{I}^{1}+1}\\ .\\ .\\ .\\ i_{m_{I}^{p}} \end{array}\right]}}=\underset{S_{I}}{\underbrace{\left[\begin{array}{cc} 1 & \Delta \psi_{m_{I}^{1}}\\ 1 & \Delta \psi_{m_{I}^{1}+1}\\ . & .\\ . & .\\ . & .\\ 1 & \Delta \psi_{m_{I}^{p}} \end{array}\right]}}\underset{O_{I}}{\underbrace{\left[\begin{array}{c} {\tilde{i}}_{m_{I}^{1}}\\ {\left(L_{k_{I}}\right)}^{-1} \end{array}\right]}}$$

where $I_{mI}\in R^{m_{I}^{1}-m_{I}^{p}+1}$ is the measurement vector of the current in the charging phase, $S_{I}\in R^{2*(m_{I}^{1}-m_{I}^{p}+1)}$ denotes the regression matrix and $O_{I}\in R^{2}$ is the output vector to be determined. To improve the robustness of the estimator and decrease the measurement errors of the current, the estimation ${\tilde{i}}_{m_{I}^{1}}$ in vector $O_{I}$ can replace the initial value $i_{m_{I}^{1}}$ in Equation (14). The approximation ${\hat{O}}_{I}$ of the output vector $O_{I}$ in the least squares sense is given by

(15) $${\hat{O}}_{I}={\left[{\left(S_{I}\right)}^{T}S_{I}\right]}^{-1}{\left(S_{I}\right)}^{T}I_{mI}.$$

The estimations for the initial current value ${\hat{\tilde{i}}}_{m_{I}^{1}}={\hat{O}}_{I}^{1}$ and the inductance ${\hat{L}}_{k_{I}}=T_{sp}/{\hat{O}}_{I}^{2}$ are obtained in charging phase I. In the same approach, the estimations for the initial value of the current ${\hat{\tilde{i}}}_{m_{II}^{1}}={\hat{O}}_{II}^{1}$ and the inductance ${\hat{L}}_{k_{II}}=T_{sp}/{\hat{O}}_{II}^{2}$ can be obtained in discharging phase II.

If the current ripple is almost triangular and the time derivative of inductance ${\dot{L}}_{k_{I}}$ or ${\dot{L}}_{k_{II}}$ is constant within $\left[t_{I}^{1},t_{I}^{p}\right]$ or $\left[t_{II}^{1},t_{II}^{q}\right]$, respectively, the average value of the inductance is defined as

(16) $$\left\{ \begin{array}{l} {\bar{L}}_{k_{I}}={\hat{L}}_{k_{I}}-{\dot{L}}_{k_{I}}\frac{\Delta t_{I}}{\Delta i_{I}}{\bar{i}}_{I}\\ {\bar{L}}_{k_{II}}={\hat{L}}_{k_{II}}-{\dot{L}}_{k_{II}}\frac{\Delta t_{II}}{\Delta i_{II}}{\bar{i}}_{II} \end{array}\right.,$$

where $\frac{\Delta t_{I}}{\Delta i_{I}}=\frac{t(t_{I}^{p})-t(t_{I}^{1})}{i(t_{I}^{p})-i(t_{I}^{1})}$ and $\frac{\Delta t_{II}}{\Delta i_{II}}=\frac{t(t_{II}^{q})-t(t_{II}^{1})}{i(t_{II}^{q})-i(t_{II}^{1})}$ are the reciprocals of the current derivative Δi/Δt, ${\dot{L}}_{k_{I}}={\bar{L}}_{k_{I}}-{\hat{L}}_{k_{I}}$ and ${\dot{L}}_{k_{II}}={\bar{L}}_{k_{II}}-{\hat{L}}_{k_{II}}$ are the estimation errors and ${\bar{i}}_{I}=\frac{1}{p}\sum_{j=1}^{p}i_{I}^{j}$ and ${\bar{i}}_{II}=\frac{1}{q}\sum_{j=1}^{q}i_{II}^{j}$ are the average current of the charging and discharging phases, respectively. The average value of the inductance in the *k*th PWM cycle can be yielded as

(17) $${\bar{L}}_{k}=\frac{{\bar{L}}_{k_{I}}\Delta i_{I}{\bar{i}}_{II}\Delta t_{II}-{\bar{L}}_{k_{II}}\Delta i_{II}{\bar{i}}_{I}\Delta t_{I}}{\Delta i_{I}{\bar{i}}_{II}\Delta t_{II}-\Delta i_{II}{\bar{i}}_{I}\Delta t_{I}}.$$

If $\Delta t_{I}=\Delta t_{II}=T_{sp}$ and $\Delta i_{I}=-\Delta i_{II}$ are assumed, Equation (17) can be simplified to

(18) $${\bar{L}}_{k}=\frac{{\bar{L}}_{k_{I}}+{\bar{L}}_{k_{II}}}{2}.$$

Estimation for the rotor position is derived from Equation (18) as

(19) $${\hat{x}}_{g1}=K_{N}/{\hat{\bar{L}}}_{k}.$$

DCE-LSM obtains better estimation precision of the rotor position [31]. However, its operation is limited in industrial applications due to the time consumed by the algorithm and the electronic circuit costs. The LSM algorithm is essentially a position estimation algorithm using the average inductance in every PWM cycle. Therefore, seeking an equivalent LSM algorithm and a solution with cheaper hardware cost is necessary.

### 2.4. SS-DCE Algorithm

As shown in Figure 8, if the working current is sampled at the instant in the *k*th PWM cycle,

(20) $$t_{ks}=kT_{s}+(1+\alpha_{k})T_{s}/2,$$

Then

(21) $$\sum_{n=1}^{\infty }\frac{4V\left|\sin(n\pi \alpha_{k})\right|}{n^{2}\pi \omega_{s}L}\cos(n\omega_{s}t-n\pi \alpha_{k})=0.$$

Equation (8) can be rewritten as

(22) $$\begin{array}{l} i_{ks}(k)=i_{k}(t_{ks})=\frac{V(2\alpha_{0}-1)}{R}+\frac{2V\alpha_{mk}\cos(\omega_{c}t_{ks})}{\sqrt{R^{2}+{\left(\omega_{c}L\right)}^{2}}}\\ i_{ks}(k)=I_{0}+I_{km}\cos(\omega_{c}t_{ks}) \end{array}$$

where $T_{s}$ is the switching period, $I_{0}$ is the bias current $\frac{V(2\alpha_{0}-1)}{R}$, $\alpha_{mk}$ is the quantity variation, $t_{ks}$ is the sampling instant and $I_{km}$ is the control current amplitude $\frac{2V\alpha_{mk}}{\sqrt{R^{2}+{\left(\omega_{c}L\right)}^{2}}}$. Assuming that the control frequency of rotor $\omega_{c}$ is far less than the carrier frequency $\omega_{s}$, we can ensure that $\cos(\omega_{c}t_{ks})\neq 0$. Then, the inductive impedance of the coil can be obtained as

(23) $$L_{ks}=\sqrt{{\left(\frac{2V\alpha_{mk}\cos(\omega_{c}t_{ks})}{i_{ks}(k)-I_{0}}\right)}^{2}-R^{2}}.$$

Therefore, the rotor position can be depicted as

(24) $${\hat{x}}_{g2}=K_{N}/L_{ks}.$$

Furthermore, by considering the equations of the *k*th cycle and the (*k* − 1)th cycle from Equation (22), we can yield the differential current:

(25) $$i_{ks}(k)-i_{(k-1)s}(k-1)=\frac{{\hat{x}}_{g2,k}}{K_{N}}\left[VT_{s}(2\alpha_{k}-1)\right].$$

Consequently, the estimation for the rotor position is

(26) $${\hat{x}}_{g2,k}=\frac{VT_{s}(2\alpha_{k}-1)}{K_{N}\left[i_{ks}(k)-i_{(k-1)s}(k-1)\right]}.$$

In Figure 8, during one switching cycle in the time interval [*t*_k_, *t*_k+1_], two instants, $t_{k}^{+}$ and $t_{k}^{-}$, are defined as the moments at which the voltage changes from −*V* to +*V* and conversely, respectively. Since the duty cycle for this period is at fixed $\alpha_{k}$ and the symmetrical triangle wave period is *T_s_*, we can obtain $t_{k}^{-}-t_{k}^{+}\equiv \alpha_{k}T_{s}$ and $t_{ks}$ as

(27) $$t_{ks}=t_{k}^{+}+T_{s}-T_{s}(1-\alpha_{k})/2=kT_{s}+T_{s}(1+\alpha_{k})/2.$$

Assuming $\alpha_{k}=50\%$, Equation (25) can be rewritten as

(28) $$i_{k}(k)=i_{k-1}(k-1).$$

Briefly, from the above conclusions, the self-sensing SS-DCE process is shorter than the other estimator processes in this study because the signal conditioning the electronic circuits can be canceled, and the operation of the position estimation algorithm is simple. In the digital signal processor, the duty cycle can be provided by the controller, and the symmetrical triangle wave is easy to generate in FPGA (field programmable gate array) or other digital signal process chips from Texas Instruments Incorporated.

Equations (24) and (26) are both formulas of the rotor position. Equation (26) is limited since the current change of the steady state is smaller than that of the transient state. Equation (26) may overflow if the AD convector cannot distinguish the adjacent sampling current. Another issue may occur when $i_{k}(k)=i_{k-1}(k-1)$ and, simultaneously, $\alpha_{k}=50\%$. That is, the SS-DCE estimator will fail if the force on the rotor is constant. Consequently, Equation (24) must dominate in the running system and Equation (26) must somehow supplement, e.g., the rotor uprising or landing.

Equation (28) can be a holder of the sampling current, which is obtained with the 50% duty cycle. Furthermore, the coil current is kept unchanged during this time, which helps coordinate with the mechanical system. Upon insertion of one or more 50% control cycles, the rotor vibration would be regulated.

## 3. Results and Discussion

### 3.1. Experimental Platform

**Figure 9:**
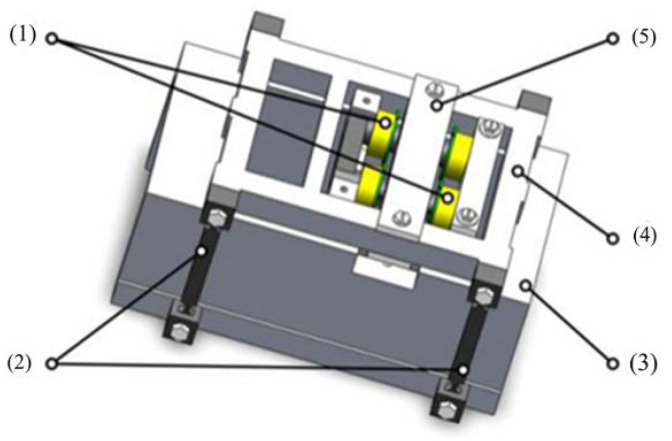
Micromotion displacement platform system. (1) electromagnetic coil; (2) flexible hinge; (3) pedestal; (4) truss; (5) lower rotor.

To verify the effectiveness of the proposed method, a platform for the experimental AMB was developed herein based on SS-DCE and dual closed-loop control. The experimental platform comprised a controller based on digital signal processor TMS320F2812, a micropositioning platform, current sensor LEM HX-05P, switching PAs and a referring position sensor HZ-891. The micromotion displacement platform was composed of a base, a truss and a flexible hinge. The double-parallel four-bar flexible hinge supported the truss, and the rotor was embedded and fixed on the truss, as shown in Figure 9. The double-parallel four-bar flexible hinge mechanism adopted a symmetrical design on both sides. When the displacement in the horizontal direction was caused by force, the displacements on both sides of the hinge in the vertical direction were equal, which produced strict translation. This could ensure the stability of the single-DOF magnetic bearing in the vertical direction.

**Figure 10:**
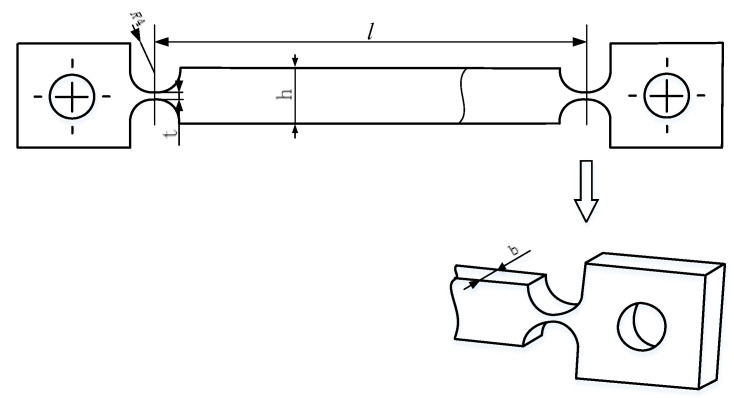
Straight circular-cut flexible hinge structure.

As shown in Figure 10, the flexible hinge adopted a straight circular-cut flexible hinge structure design, and the material was carbon steel. The specific parameters are shown in Table 1:

The rigidity formula of the flexible hinge’s four-bar mechanism is

(29) $$K=\frac{8Ebt^{5/2}}{9\pi R^{1/2}l^{2}}.$$

Calculated with specific parameters, *K* = 0.0816 N/m. Since the micromotion displacement platform adopts the structure of a double-parallel four-bar flexible hinge, the total stiffness iwass 2 *K* = 0.1632 N/m.

**Figure 11:**
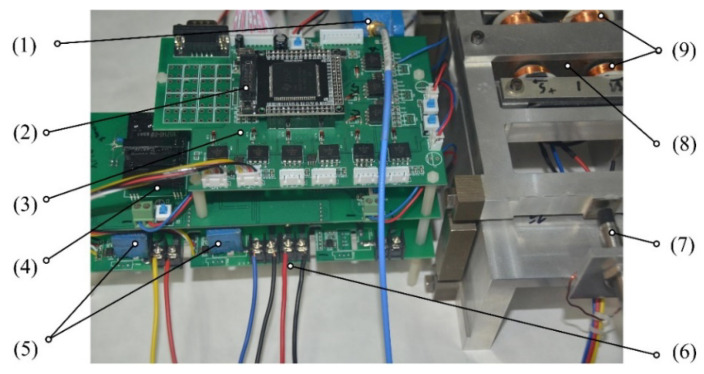
Self-sensing AMB’s experimental platform. (1) Reference sensor amplifier; (2) controller board; (3) signal process board; (4) power board; (5) PA board; (6) current sensor; (7) reference sensor HZ-891; (8) rotor; (9) electromagnets.

The experimental platform used an intelligent power module (PM10CSJ060) designed for power switching applications at 2-kHz frequencies. PAs are configured in two-state modes (±50 V) to ensure high-frequency ripples and increased the working stability of the self-sensing AMB. The built-in control circuits provided the optimum gate drive and protection for the power devices. The experimental platform entity is shown in Figure 11. In Table 2, the main parameters of the experimental self-sensing AMB are listed. The force/displacement factor *k*_x_ is calculated by [55].

(30) $$k_{x}=-\frac{\mu_{0}N^{2}AI_{0}^{2}}{2g_{0}^{3}}=-2.8\times 10^{4}N/m.$$

A vital part of SS-DCE is a synchronized-sampling event, which is implemented in the timer underflow interrupt T1UFINT/T3UFINT of the EVA/EVB of TMS320F2812. Since the interrupt occurs at instant *t_ks_*, obtaining the current synchronized with the PWM signal is not difficult.

In this study, the simulation and experimental resulted were compared with several other estimation algorithms, including the DFAD approach, DCM approach and DCE-LSM and SS-DCE position estimation approaches. The current and voltage signals were digitized via a 100-kHz A/D converter when filtered by the analog BPFs that isolate the fundamental component to improve the sampling resolution. Then, the ideal absolute value functions were implemented to detect the envelope of the fundamental component of the current and voltage, and the position information was shifted to low frequencies. LPFs can select only low-frequency baseband signals as the control signal. An FIR filter was employed in the digital signal processor.

**Figure 12:**
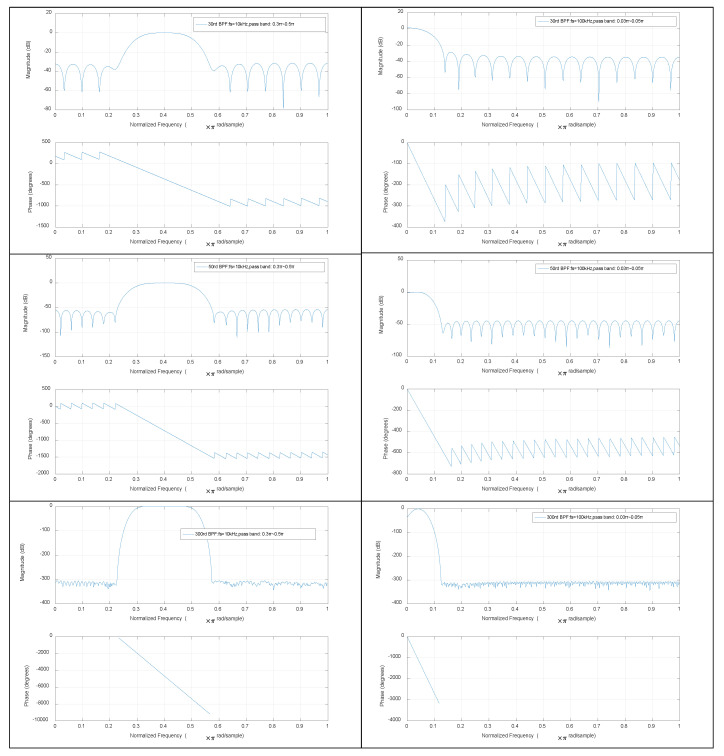
Bode plots of 30-, 50- and 300th order BPF with different sample frequencies.

**Figure 13:**
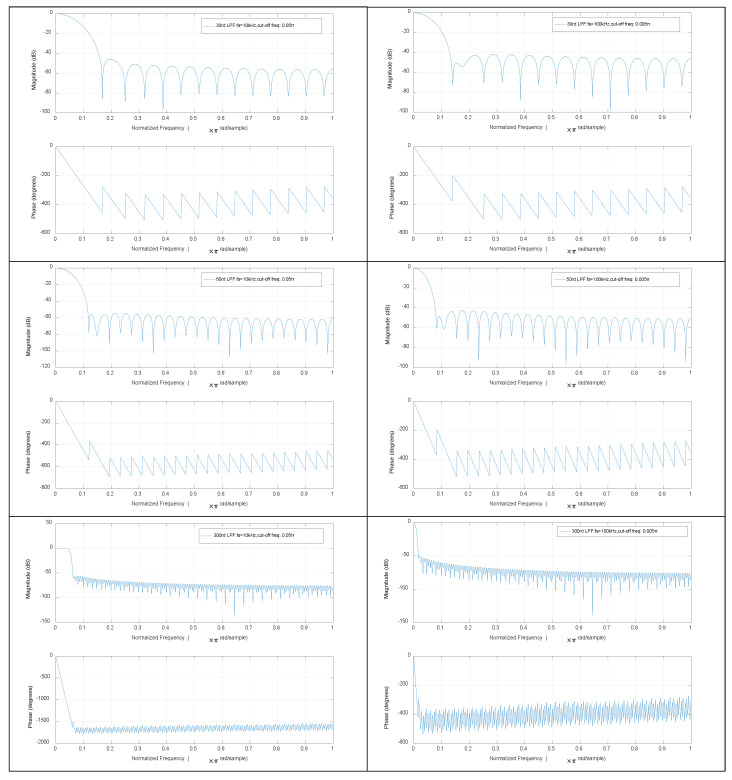
Bode plots of 30-, 50- and 300th order LPF with different sample frequencies.

**Figure 14:**
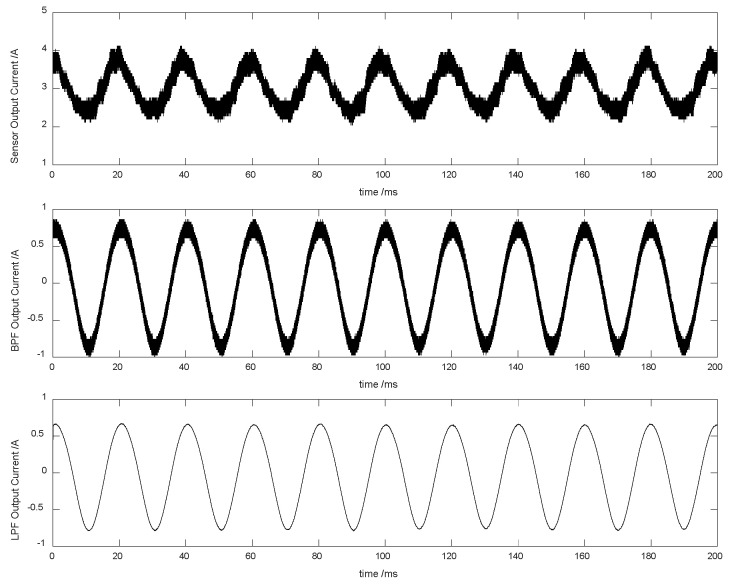
Currents of the DFAD estimator (50 Hz).

The Bode plots of BPF are shown in Figure 12. The band-pass of 30-, 50- and 300th order BPFs was 0.3–0.5π when the sample frequency fs = 10 kHz. The 30th order filter precision was the lowest level, but its phase lag was the smallest; the 300th order filter precision was the highest level, but its phase lag was the largest. When the sample frequency fs = 100 kHz, BPFs’ Bode plots were noticeably closer to the corresponding LPFs. Accordingly, BPF could be replaced by LPF in practice because the former had a low-pass effect and the fundamental ripple was just 2 kHz. The Bode plots of the last LPF were shown in Figure 13, from which similar conclusions could be drawn. The 30th order filters of BPF and LPF were qualified for the self-sensing process. The 50-Hz working currents by sensor, BPF and LPF in the DFAD estimator were shown in Figure 14. Since the power frequency was 50 Hz, the noise and disturbance of 50 Hz were obvious, and significantly, the self-sensing estimator was tested at 50 Hz.

Thereafter, the rotor position can be calculated by the quotient of the max value of the current and voltage and be compensated by the nonlinearity of magnetic material as

(31) $$1/\mu_{r}=a_{m2}B^{2}+a_{m1}B+a_{m0},$$

where $a_{m2}$ $a_{m1}$ and $a_{m0}$ are the coefficients of the quadratic polynomial that are determined via the simple experiments reported by Schammass [16], magnetic flux density B is obtained by $\mu_{0}Ni_{L}/2x_{g-1}$ and $x_{g-1}$ is the prior value of the estimator output. In Table 3, the self-sensing parameters for AMB are listed. The PD and PI controller parameters are tuned based on the extended critical proportion method. The slight difference in the estimator convergence rate stems from the control parameters. For example, the SS-DCE convergence rate when *K*_d_ = 10 and *K*_d_ = 22 is lower than that when *K*_d_ = 20 and *K*_d_ = 45; however, *K*_d_ cannot be over 68. Thus, the frequency of the first closed-loop mode of the test rig is obtained:

(32) $$\begin{array}{c} \omega_{ec}\approx \sqrt{-(K_{x}+K_{p}\cdot K_{i})/m}=1242.60\\ \mathrm{or}\;f_{ec}=\omega_{ec}/(2\pi )=197.77\mathrm{Hz} \end{array}$$

### 3.2. Simulation Results

**Figure 15:**
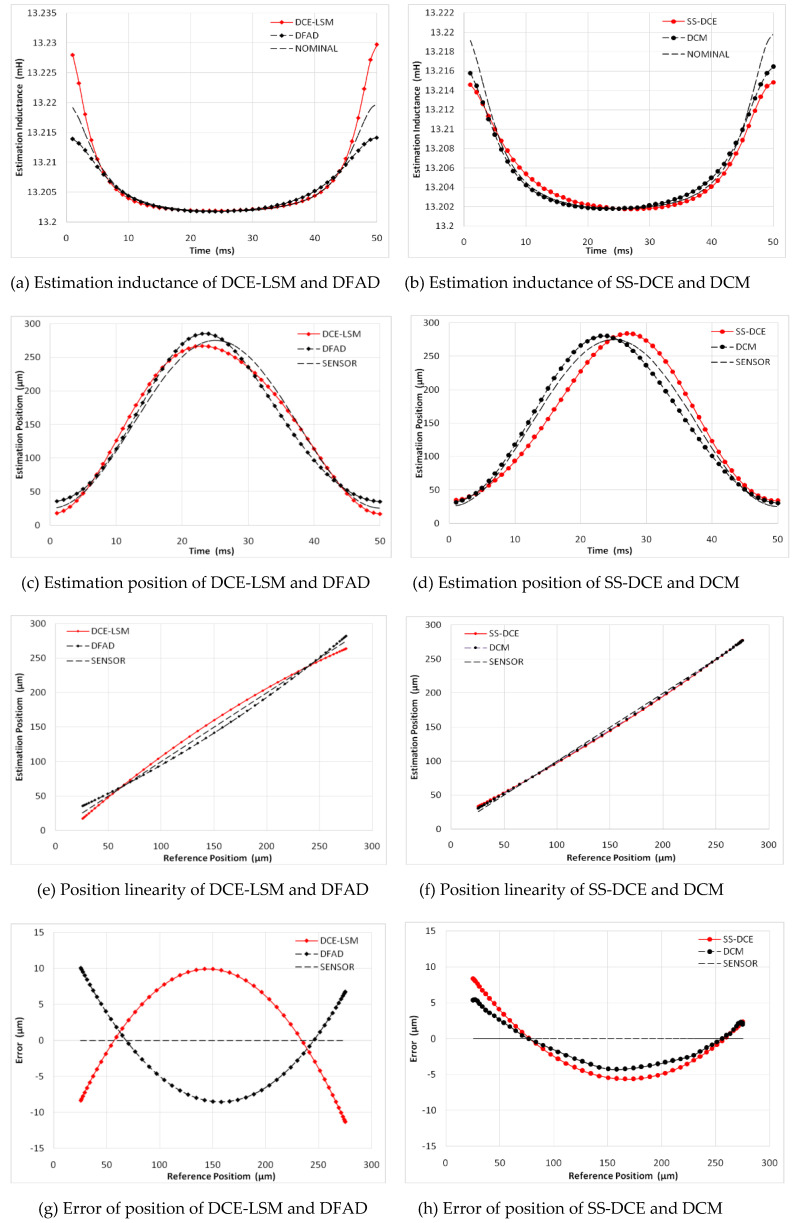
Static performance simulation results (20 Hz).

A sinusoidal excitation signal with a 10-µm peak–peak value was applied as a reference position with a frequency ranging from 20 to 200 Hz. Figure 15 shows the simulation results of the static performances at 20 Hz. In Figure 15a,b, the normalization inductances of the four estimators are depicted with the nominal inductance in a working cycle. The static simulation results of the position estimators are shown in terms of sensor linearity and the error referring to the theoretical result of the AMBs’ output. Under open-loop conditions with a constant bias current of 3.0 A, the desired position linearly varied between 50 and 250 µm. Figure 15c,d shows the estimation position compared to the ideal set value, and the linearity and error of the estimation position are shown in Figure 15e–h. As the estimation error is not discernible in Figure 15e,f, the detailed error is shown in Figure 15g,h. Since the test frequency of 20 Hz was close to the natural frequency, the output position and error of these estimators were affected by the resonance characteristic of the rotor.

**Figure 16:**
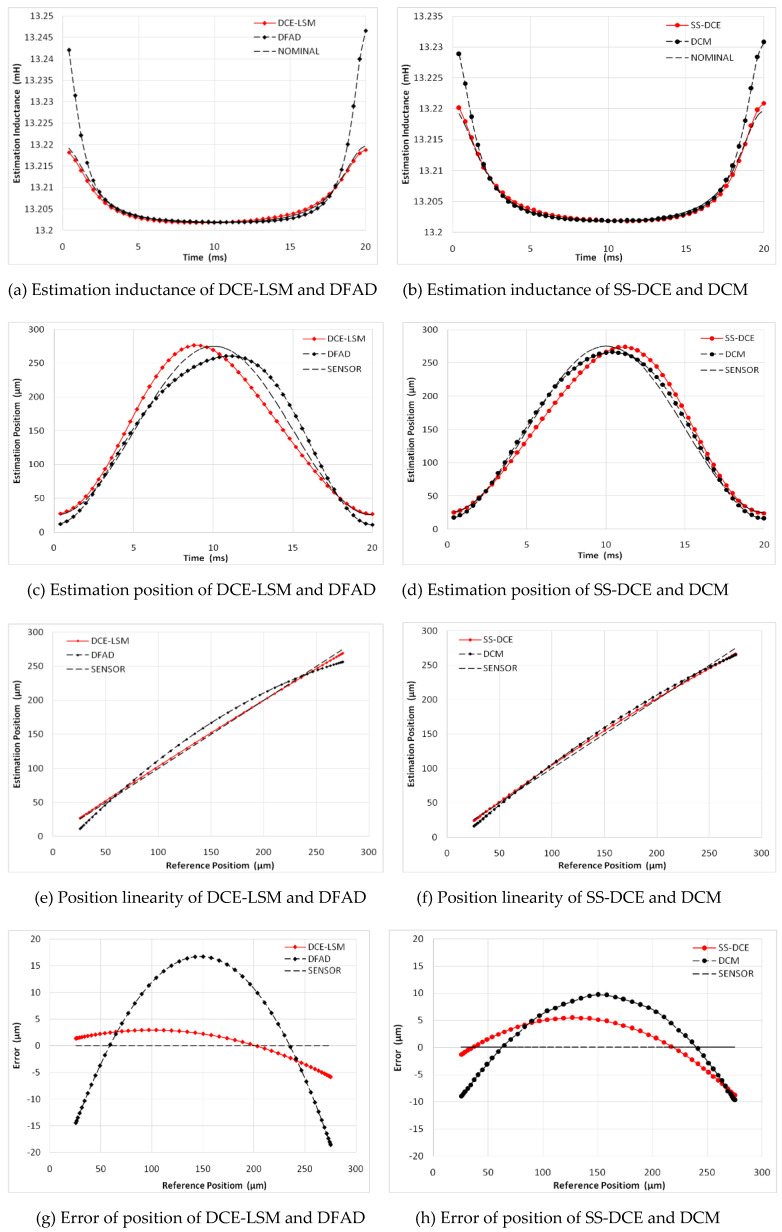
Static performance simulation results (50 Hz).

**Figure 17:**
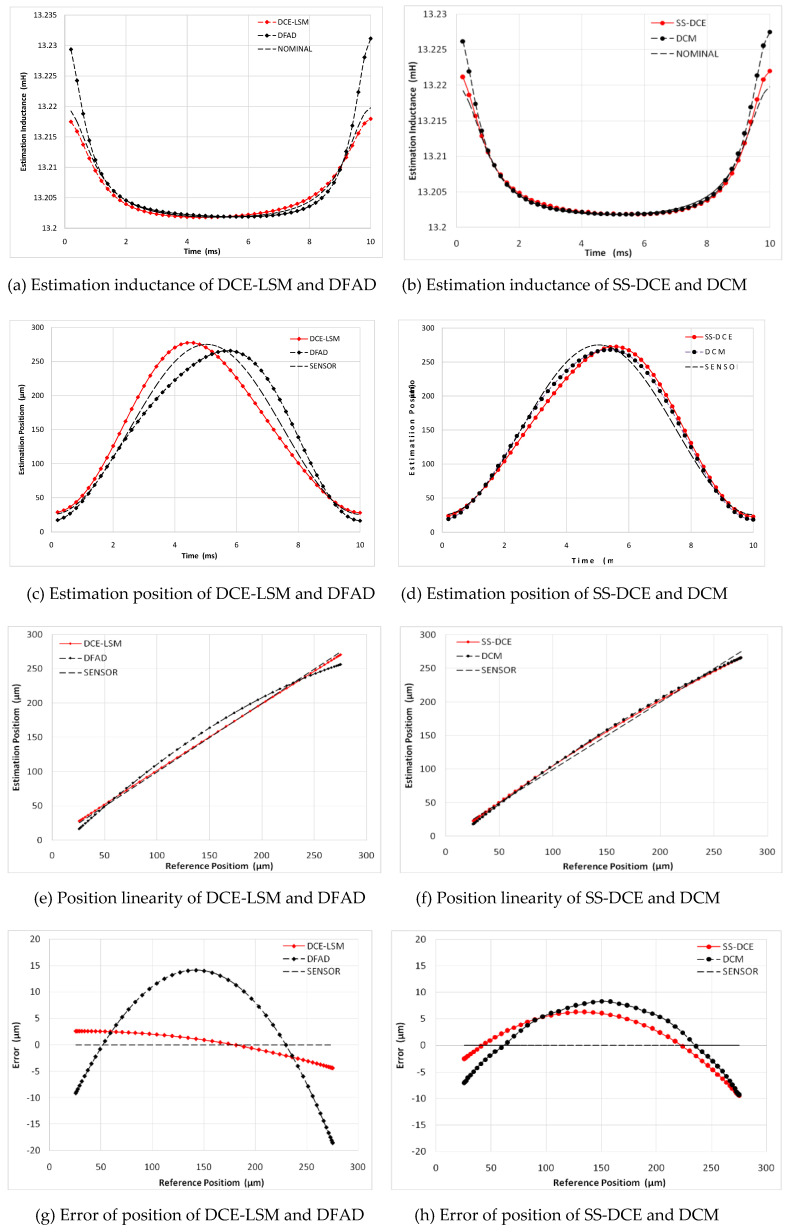
Static performance simulation results (100 Hz).

**Figure 18:**
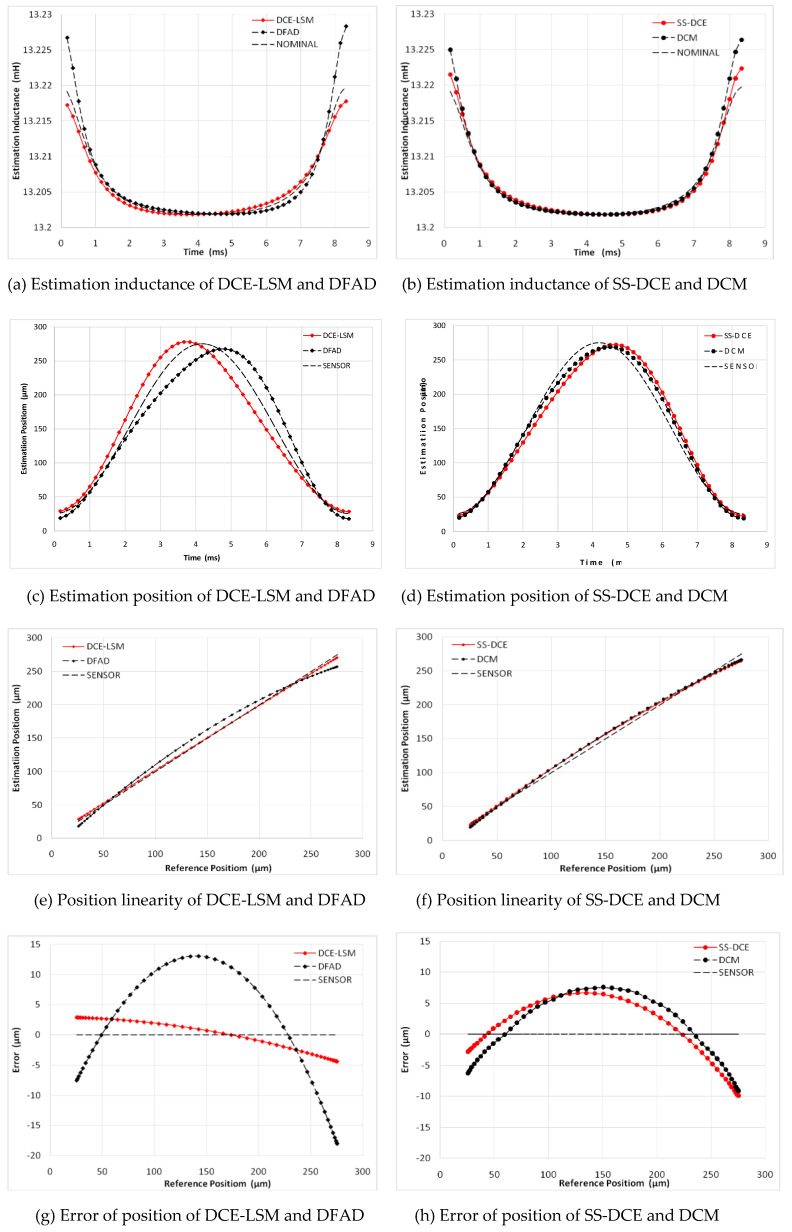
Static performance simulation results (150 Hz).

**Figure 19:**
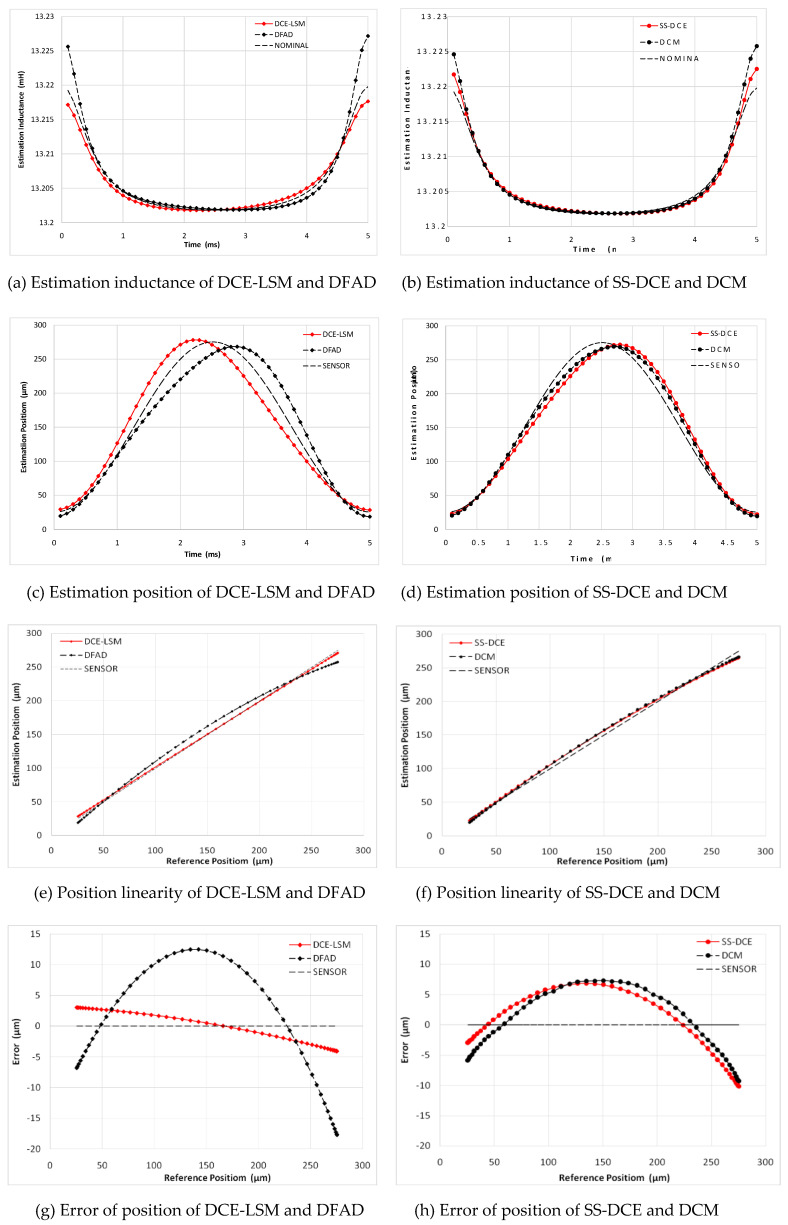
Static performance simulation results (200 Hz).

**Figure 20:**
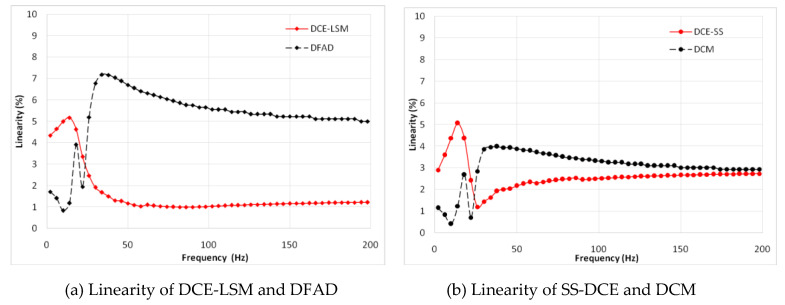
Simulation results of estimator precision.

In Figure 15, a minimum error is observed for the DCE-LSM demodulation algorithm in the 50–250 µm range. The error of the SS-DCE demodulation algorithm is equivalent to that of DCM. The DCE-LSM demodulation algorithm showed excellent linearity, but its CPU overhead was larger than that of the others. In regards to related research conclusions and a factual working status, the inductance, position, linearity and error parameters in the transition frequency band (50–200 Hz) held important significance to characterize the system performance of the self-sensing AMB. To address the matter, static performances at frequencies of 100, 150 and 200 Hz were performed, as shown in Figure 16, Figure 17, Figure 18 and Figure 19. The proposed SS-DCE method could obtain accurate position and inductance estimation results. Compared to the DCE-LSM and DFAD methods, the SS-DCE method conducted better linearity and error in the transition frequency band. In Figure 20, the results of these estimators’ precisions are outlined in the 0.1–200 Hz range. Apparently, the SS-DCE method had higher linearity precision, especially for the rising-speed frequency range.

Moreover, the above results present that both the DCE-LSM and SS-DCE outputs differed by less than 7.5 µm from the reference signal of the theoretical model in the 50–250 µm range when the test frequency was kept away from the natural frequency. This was primarily attributed to the adequate data obtained for estimating the working coil current.

**Figure 21:**
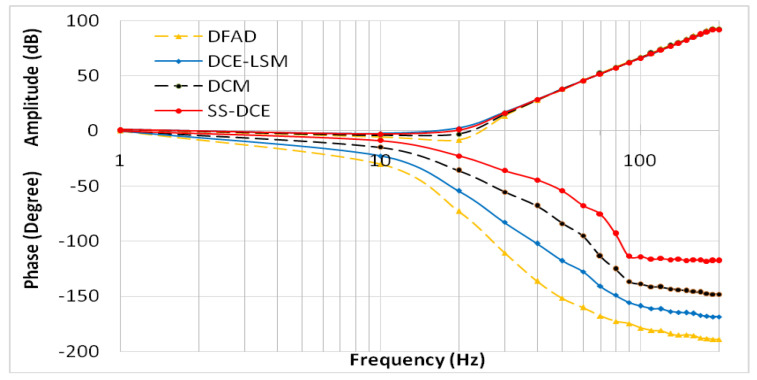
Gain and phase response of Gx(ω).

The gain and phase response of the self-sensing system are denoted as

(33) $$G(\omega )=20\log\left(X_{d}(\omega )/X_{r}(\omega )\right),$$

where $X_{d}(\omega )$ is the self-sensing output and $X_{r}(\omega )$ is the reference signal. In Figure 21, the comparative results show that the phase-shift could be clearly reduced by referring to the digital demodulation, demonstrating that the phase lag of SS-DCE was around −22° at the natural frequency, but was −62° phase margin when the rotor worked at 200 Hz.

Comparison of the simulation results of DFAD, DCM and DCE-LSM demonstrated that the estimation precision of DCE-LSM was higher than the output of the other estimators due to the sampling current accuracy and estimation algorithm. The difference between DCE-LSM and reference sensor was the smallest, and the linearity was the best. Meanwhile, the DFAD, DCM and SS-DCE estimation methods were affected by the signal data sampled once in the PWM switching cycle.

### 3.3. Experimental Results

**Figure 22:**
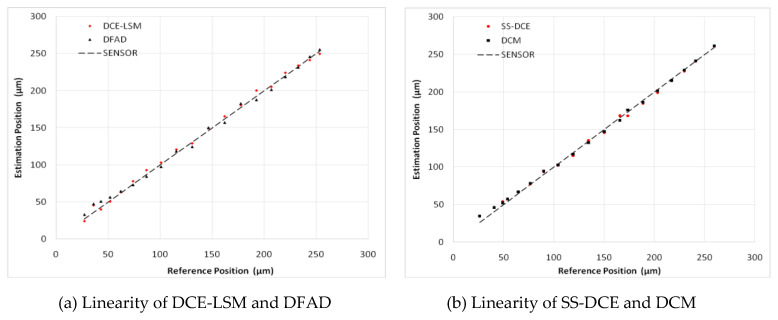
Experimental result of estimator linearity (20 Hz).

**Figure 23:**
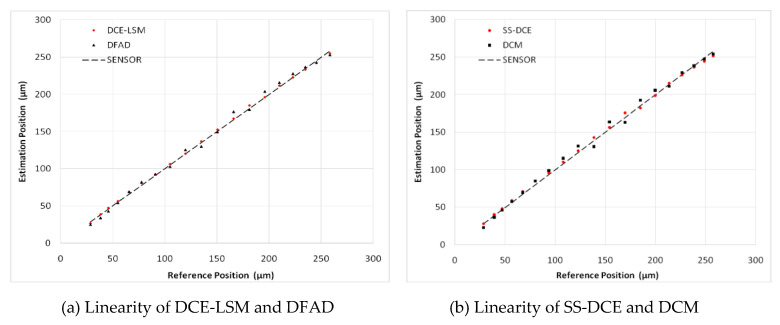
Experimental result of estimator linearity (50 Hz).

**Figure 24:**
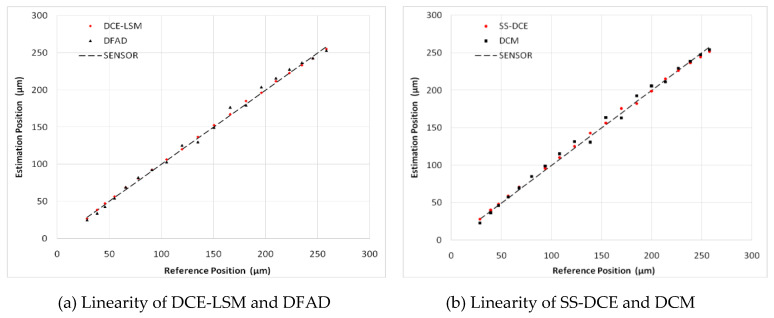
Experimental result of estimator linearity (100 Hz).

**Figure 25:**
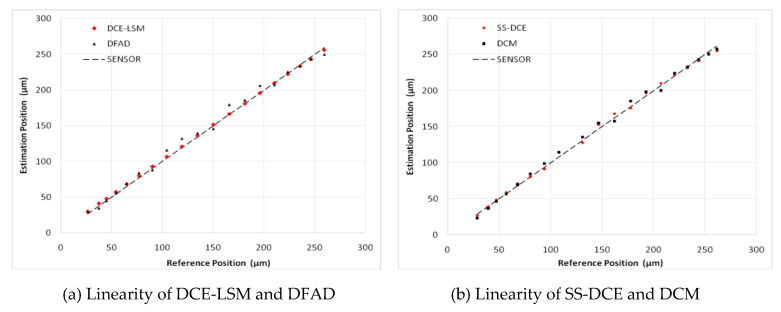
Experimental result of estimator linearity (150 Hz).

**Figure 26:**
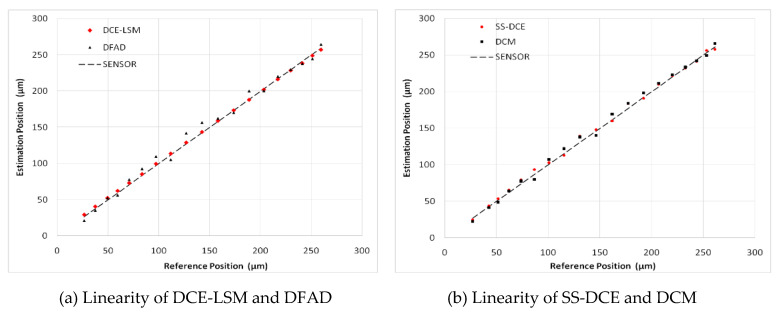
Experimental result of estimator linearity (200 Hz).

To verify the estimator and restrict the air gap of the rotor in the 50–250 μm range, the output of the position estimator was compared to that of the eddy current sensor HZ-891. The linearity test results are shown in Figure 22, Figure 23, Figure 24, Figure 25 and Figure 26.

**Figure 27:**
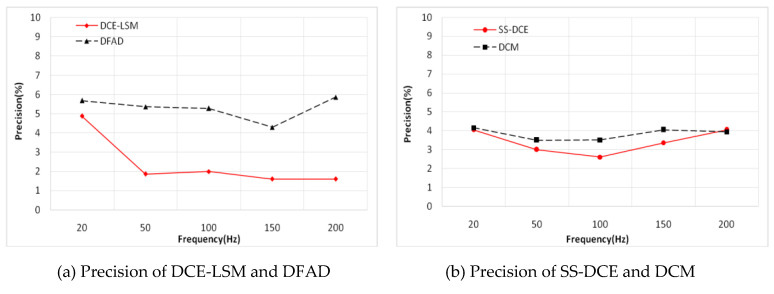
Experimental result of estimator precision.

The estimation linearity of DCE-LSM was better than that of the others, and its estimation precision was less than 2% when the test frequency was far from the natural frequency. Due to more additional phase-shifts introduced in the sensor process, the linearity of DFAD was the poorest; the estimation accuracy presented in Figure 27 is about 6%. The precision of DCM and SS-DCE basically maintained a similar level. The experimental results are slightly better than the simulation results because the rotor was dominated by the damping force of the eddy current that included the motion and electrical eddy current. This damping force was also associated with and affected the rotor vibration and position estimator reported by Yu et al. [52] and Ji et al. [53]. Moreover, the experimental results of the four estimators regarding the mass are consistent with the simulation results.

**Figure 28:**
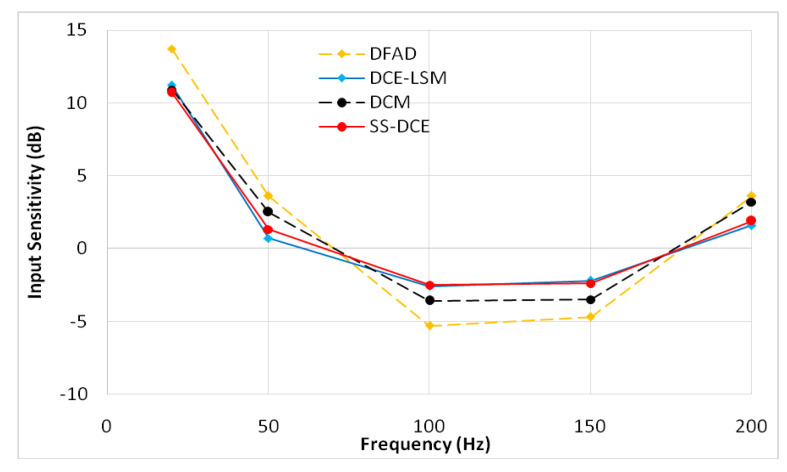
Experimental input sensitivity of estimators.

The input sensitivity analysis was operated according to ISO-14,839–3 because no robustness indicators exist for self-sensing AMBs. In Figure 28, the suspended rotor was excited by a sinusoidal signal with a 10-μm peak–peak value from 20 to 200 Hz. The results demonstrate that the input sensitivity peak value listed in Table 4 occurred when the frequency (20 Hz) was close to the natural frequency; the AMBs with SS-DCE kept the lowest sensitivity.

## 4. Conclusions

In this study, the phase shift and stability challenges introduced by demodulation filters and self-sensing algorithms were addressed with the proposed SS-DCE approach. The strengths and weaknesses of this approach were determined by comparison to four different position estimation approaches of AMBs.

A large number of filters are applied in the DFAD and DCM approaches to estimate the position obtained by the fundamental amplitude of the current and voltage. Due to the additional phase-shift introduced by these filters, the system bandwidth is limited, and the achievable stability margin is reduced. A novel method for the position estimation of self-sensing AMBs with a PWM-controlled magnetic suspension system was proposed based on a detailed mathematical model that is a viable description of the effect of switching PAs on the time evolution of the rotor current and position. Based on an analysis of the charging/discharging phases of the coil, the error induced by both a working value of the electric resistance and the dynamic inductance was considered.

Compared to these different methods, the SS-DCE approach had great advantages regarding its estimator. When the test frequency (20 Hz) was close to the natural frequency, the error and precision of all estimators achieved the best performance. When the test frequency was far from the natural frequency, the DCE-LSM estimation’s linearity was better than that of the others; the simulation result was less than 2%, and the corresponding experimental result was about 2% in the 50–250 μm range. Due to more additional phase-shifts introduced in the sensor process, the linearity of DFAD was the poorest, and the estimation linearity presented was about 6%. Similar linearity between SS-DCE and DCM was less than 4%. However, SS-DCE had an excellent dynamics performance in the open-loop state, which was evaluated by a frequency response analysis; a 158° phase margin was shown as at the natural frequency of 19.26 Hz, and the sensitivity peak is 10.7 dB in the 50–250 μm range.

According to the simulation and experimental results, the precision of the amplitude demodulation approach was lower because of the neglected dynamic estimation of inductance and the insufficient signal data in the estimation process. DCE-LSM considered the inductance dynamics and optimized the calculation algorithm, but its calculation overhead was too excessive, increasing the hardware and software costs. Thus, SS-DCE was derived, wherein an emphasis was placed on a special description of the influence of the switching amplifier on the duty cycle evolution of the current. Furthermore, the robustness limitations of self-sensing magnetic bearings are concerned in the commercial application. A limitation of this study is that it only applies to self-sensing AMBs using bistate switching PA.

## Figures and Tables

**Table 1:** Flexible hinge parameters for the AMB.

Parameters	Values
Elastic modulus: *E/Gpa*	196.2
Width of flexible hinge: *b/mm*	14
Height of flexible hinge: *h/mm*	9
Minimum thickness of flexible hinge: *t/mm*	1
Notch radius: *R/mm*	4
Mechanism length: *l/mm*	69

**Table 2:** Parameters of single-DOF AMB.

Parameters	Values
Single magnetic pole area: *A*/cm^2^	6.16
Coil number: *N*/turns	50.00
Initial gap: *g*_0_/μm	6.76 × 10^2^
Rotor mass: *m*/kg	1.93
Relative permeability: μ_r_	2.30 × 10^3^
Coil resistance: *R*/Ω	0.50
Nominal inductance: *L*_0_/mH	13.20
PWM frequency: *f_s_*/kHz	2.00
Sampling frequency of DFAD/DCM/LSM:/kHz	100.00
Bias current: *I*_0_/A	3.00
Force/displacement factor: *k*_x_/(N/m)	−2.8 × 10^4^
Natural frequency of test rig:/Hz	19.26

**Table 3:** Self-sensing parameters of the AMB.

Estimators	Positions (PD)		PAs (PI)		Compensators		
	*K*_p_ (A/mm)	*K*_d_ (A·s/m)	*K*_p_	*K*_I_	a_m2_	a_m1_	a_m0_
DFAD	10	22	0.2	0.01	5.51 × 10^5^	−1.92 × 10^5^	4.10 × 10^6^
DCM	20	38	0.5	0.01	−2.50 × 10^5^	1.03 × 10^5^	3.78 × 10^6^
DCE-LSM	20	37	0.5	0.012	−2.50 × 10^5^	1.03 × 10^5^	3.78 × 10^6^
SS-DCE	20	45	0.5	0.012	−2.50 × 10^5^	1.03 × 10^5^	3.78 × 10^6^
Sensor	15	32	0.2	0.01	–	–	–

**Table 4:** Summary of the input sensitivity of the proposed estimators.

Estimator	DFAD	DCE-LSM	DCM	SS-DCE
Sensitivity Peak	13.7	11.2	10.9	10.7
